# Six Extensively Drug-Resistant Bacteria in an Injured Soldier, Ukraine

**DOI:** 10.3201/eid2908.230567

**Published:** 2023-08

**Authors:** Patrick T. Mc Gann, Francois Lebreton, Brendan T. Jones, Henry D. Dao, Melissa J. Martin, Messiah J. Nelson, Ting Luo, Andrew C. Wyatt, Jason R. Smedberg, Joanna M. Kettlewell, Brain M. Cohee, Joshua S. Hawley-Molloy, Jason W. Bennett

**Affiliations:** Multidrug-Resistant Organism Repository and Surveillance Network, Walter Reed Army Institute of Research, Silver Spring, Maryland, USA (P.T. Mc Gann, F. LeBreton, B.T. Jones, H.D. Dao, M.J. Martin, M.J. Nelson, T. Luo, J.W. Bennett);; Landstuhl Regional Medical Center, Landstuhl, Germany (A.C. Wyatt, J.R. Smedberg, J.M. Kettlewell, J.S. Hawley-Malloy);; 512th Field Hospital, Rhine Ordinance Barracks, Germany (B.M. Cohee)

**Keywords:** antimicrobial resistance, extensively drug-resistant bacteria, Ukraine, *Acinetobacter baumannii*, *Klebsiella pneumoniae*, *Enterococcus faecium*, *Pseudomonas aeruginosa*, XDR, antibiotic resistance, hypervirulence, bacteria

## Abstract

Blood and surveillance cultures from an injured service member from Ukraine grew *Acinetobacter baumannii*, *Klebsiella pneumoniae*, *Enterococcus faecium*, and 3 distinct *Pseudomonas aeruginosa* strains. Isolates were nonsusceptible to most antibiotics and carried an array of antibiotic resistant genes, including carbapenemases (*bla*_IMP-1_, *bla*_NDM-1_, *bla*_OXA-23_, *bla*_OXA-48_, *bla*_OXA-72_) and 16S methyltransferases (*armA* and *rmtB4*).

The ongiong conflict in Ukraine has placed extraordinary pressure on medical infrastructure and health delivery services in the region (*1*). Previous reports from Eastern Ukraine have noted the emergence of multidrug-resistant (MDR) *Acinetobacter baumanii*, *Pseudomonas aeruginosa*, and Enterobacterales infections during hospitalization (*2*). Those strains encompassed a variety of clonal lineages, with many carrying carbapenemases, extended-spectrum β-lactamases (ESBLs), and 16S methyltransferases (*2*,*3*). We describe the isolation of 6 extensively drug-resistant (XDR) organisms from a single soldier from Ukraine.

A man in his mid-50s suffered multiple traumatic injuries after a vehicle fire, including full-thickness burns covering 60% of his total body surface. He was initially treated in a medical facility near Dnipro, Ukraine, before being transferred to a hospital in Kyiv, Ukraine, where healthcare practitioners performed burn wound debridement and escharotomies. Thereafter, the patient was transported to a US military hospital in Germany, where doctors obtained blood, urine, respiratory, and peri-rectal surveillance cultures. Surveillance cultures grew *A. baumannii*, *Enterococcus faecium*, *Klebsiella pneumoniae,* and 2 distinct morphologies of *P. aeruginosa*. Blood cultures grew a third *P. aeruginosa* (Table). By using the Vitek 2 automated system (bioMérieux, https://www.biomerieux.com), the gram-negative organisms were found to be nonsusceptible to almost every antibiotic tested (Appendix Table 1), with the exception of *A. baumannii*, which was susceptible to tetracycline (MIC 2 µg/mL). The *E. faecium* was nonsusceptible to vancomycin. Researchers used a customized Sensititer panel (Thermo Scientific, https://www.thermofisher.com) to test the gram-negative organisms against colistin, eravacycline, imipenem/relebactam, meropenem/vaborbactam, omadacycline, and plazomicin; they used disk diffusion (Hardy Diagnostics, https://hardydiagnostics.com) to test against cefiderocol (Appendix Table 1). Researchers performed whole-genome sequencing of all isolates by using an Illumina Miseq and the MiSeq Reagent Kit version 3 (600 cycles, 2 × 300 bp) (Illumina, https://www.illumina.com). 

**Table:** Characteristics of 6 isolates cultured from an injured service member from Ukraine*

MRSN ID	Species	ST†	Antimicrobial resistance genes‡
110819	*Acinetobacter baumannii*	78	*aph(3′)-Via*, *aac(6')-Ian*, ***armA***, *aadA5*, *ant(3′′)-IIa*, ***bla*_OXA-23_**, ***bla*_OXA-72_**, *bla*_OXA-90_, *bla*_ADC-152_, ***bla*_CTX-M-115_**, bla_CARB-16_, *catA1*, *mph(E)*, *msr(E)*, *sul1*, *sul2*
110818	*Pseudomonas aeruginosa*	357	*aac(6')-Il*, *aph(3′)-IIb*, *aadA1*, *bla*_OXA-10_, *bla*_OXA-846_, *bla*_PDC-11_, ***bla*_VEB-9_**, *catB7*, *sul1*, *tet(A)*, *dfrB2*
110817	*P. aeruginosa*	773	*aph(3′)-IIb*, *aadA11*, ***bla*_NDM-1_**, *bla*_PDC-16_, *bla*_OXA-395_, *catB7*, *qnrVC1*, ***rmtB4***, *sul1*, *tet(G)*
110606§	*P. aeruginosa*	1047	*aac(6')-Ib3*, *aph(3′)-IIb*, *aph(3′′)-Ib*, *aph(**6**)-Id*, ***bla_IMP-1_***, *bla*_OXA-10_, *bla*_OXA-488_, *bla*_PDC-12,_ *catB7*, *sul1*
110821	*Klebsiella pneumoniae*	395	*aac(6')-Ib-cr5*, *aph(3′)-VI*, *ant(2′′)-Ia*, *aadA1*, ***armA***, ***bla*_NDM-1_**, ***bla*_OXA-48_**, ***bla*_CTX-M-15_,** *bla*_OXA-1_, *bla*_SHV-11_, *bla*_TEM-1_, *catA1*, *dfrA1*, *dfrA5*, *fosA*, *mph(A)*, *mph(E)*, *msr(E)*, *oqxA*, *oqxB*, *qnrS1*, *sul1*, *sul2*, *tet(A)*
110820	*Enterococcus faecium*	117	*aac(6')-Ie*, *aacA-ENT1*, *aph(2′′)-Ia*, *catA7*, *dfrG*, *erm(B)*, *msr(C)*, ***vanA*** (operon)

*Bold indicates high-impact genes. MRSN, The Multidrug-Resistant Organism Repository and Surveillance Network; ID, identification; ST, sequence type.

†In silico–derived multilocus STs.

‡In silico–derived antimicrobial resistance gene content generated using MIGHT, a customized script wrapping ARIBA (https://github.com/sanger-pathogens/ariba) and AMRfinder (https://github.com/ncbi/amr).

§Blood culture isolate.

The *K. pneumoniae* isolate, designated MRSN 110821, was nonsusceptible to every antibiotic tested (Appendix Table 1). Testing identified 24 antimicrobial resistance genes, including the carbapenemases *bla*_NDM-1_ and *bla*_OXA-48_, the RMTase *armA*, and the ESBL *bla*_CTX-M-15_ (Table). Five plasmid replicons were identified (Appendix Table 2), but long-read sequencing is underway to better understand the plasmid structure (data not shown). Colistin resistance likely resulted from a previously characterized E82K mutation in the 2-component transcriptional regulator PhoP (*4*). Cefiderocol resistance could be linked to mutations in the outer membrane protein OmpK36 combined with NDM (*5*). The isolate also carried several hypervirulence genetic markers, including *ybt*16 (yersiniabactin siderophore), *iuc*1 (aerobactin), and *rmpADC*/*rmpA2* (mucoviscosity and capsule).

The isolate belonged to clade B1 of the clonal lineage sequence type (ST) 395 (*6*) and was K-antigen capsular biosynthesis loci, K39, and O-antigen type O2 variant 1 (O2v1). ST395 was first described in France in 2010, and carbapenemase-producing strains are increasingly being reported across Europe (*6*). We downloaded all clade B1 ST395 isolates from Pathogenwatch (https://pathogen.watch) and constructed a phylogenetic tree (Figure). We included 3 ST395 genomes identified by Sandfort et al, which they cultured from patients from Ukraine who were hospitalized in Germany (*7*). Of note, MRSN 110821 was separated by just 20 single nucleotide polymorphisms from NRZ-78043a from the Sandfort study and by just 19 from NRZ-78056b from that same study (Figure). Those 3 isolates clustered more broadly with isolates from Russia and Finland (Figure), but have since acquired *armA*, *bla*_NDM-1_, and the mucoviscosity and capsule loci *rmpADC*, further increasing their antibiotic resistance profile and virulence potential.

**Figure:**
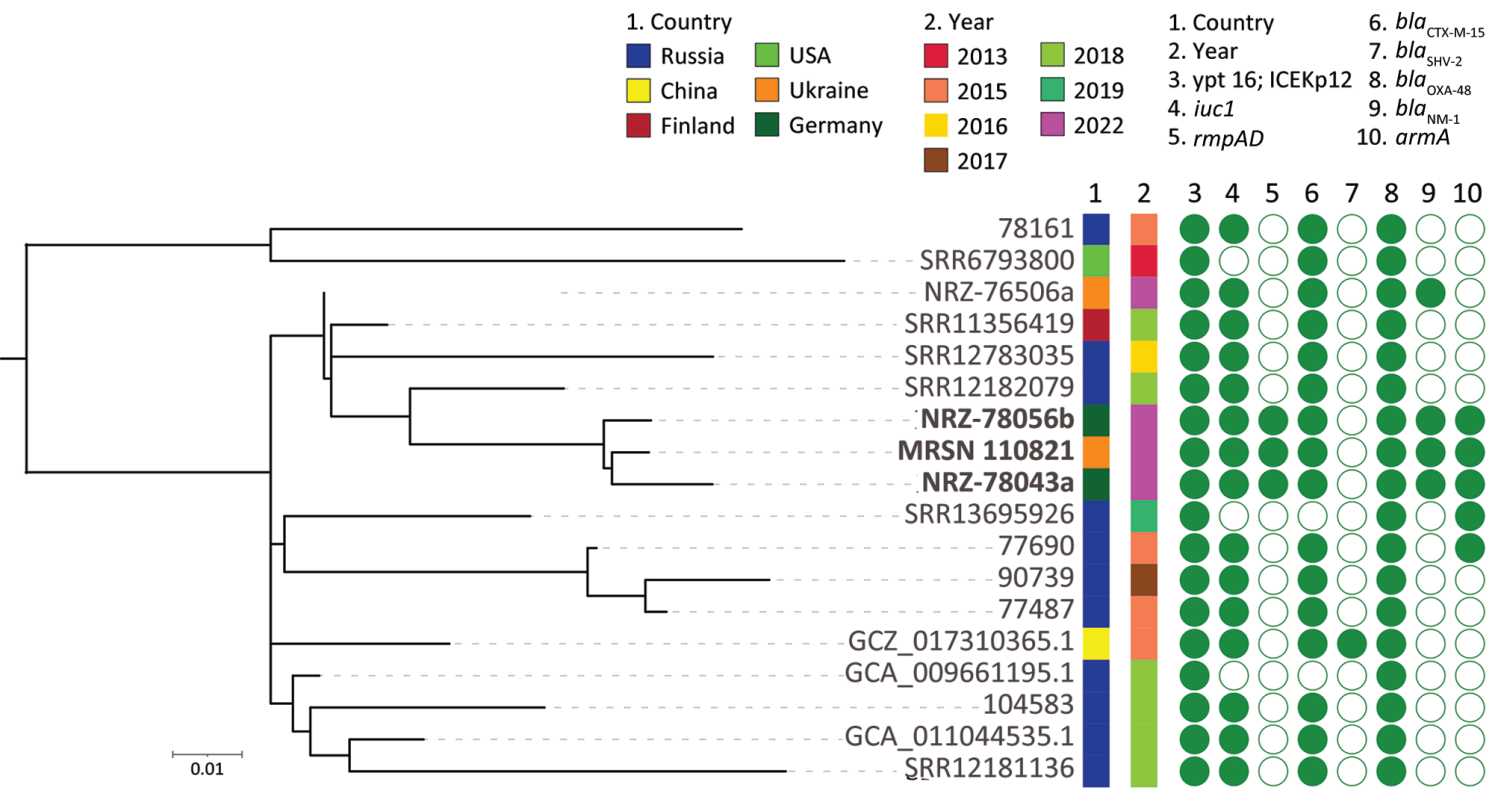
Core genome, SNP-based phylogenetic tree for *Klebsiella pneumoniae* from an injured service member from Ukraine (MRSN 110821) and 17 closely related sequence type 395 *K. pneumoniae*. In addition to MRSN 110821, the dataset included 14 subclade B2 isolates and 3 NDM-1/OXA-48–producing isolates available in public databases. Country of origin, year of collection, and presence (closed circle) or absence (open circle) of selected virulence and antimicrobial resistance genes are indicated. The midpoint was used as a root for the phylogenetic tree. *K. pneumoniae* MRSN 110821 from this study and the 2 highly related strains from Germany are highlighted in boldface. Scale bar indicates the ratio of substitutions per site for a 1,665 bp alignment of variable sites in the core genomes of the 18 strains.

We found *A. baumannii* MRSN 110819 to be resistant to all antibiotics except cefiderocol, colistin, eravacycline, and omadacycline (Appendix Table 1). The isolate carried 18 AMR genes, including the RMTase *armA*, the ESBL *bla*_CTX-M-115_, and 2 carbapenemases, *bla*_OXA-23_ and *bla*_OXA-72_ (Table). The isolate was assigned to ST78, a clonal group known as the Italian clone because it emerged in Italy in the mid-2000s (*8*). This clonal group has also been identified in war wounds of service members from Ukraine during the earlier conflict in Eastern Ukraine (*2*).

The 3 *P. aeruginosa* isolates belonged to 3 distinct strains (Table). All 3 isolates had high MICs to 20 of the 23 antibiotics tested (Appendix Table 1). Only colistin and cefiderocol appeared effective in vitro, although MRSN 110818 was susceptible to imipenem/relebactam using US Food and Drug Administration breakpoints (MIC 2 mg/L). All 3 carried carbapenemases, ESBLs, and 16S methyltransferases (Table). MRSN 110818 and 110817 belonged to well-known (ST357) and emerging (ST773) epidemic, high-risk clones that are increasingly associated with horizontally acquired β-lactamases (*9*). The single blood isolate was assigned to ST1047.

*E. faecium* MRSN 110820 carried 8 AMR genes, including the *vanA* operon (Table). The strain was assigned to ST117, a member of clonal complex 78.

Gaps in such services as infection control, caused by limited resources and personnel, are exacerbating the transmission of MDR organisms in Ukraine. As a result, healthcare networks in Europe now consider prior hospitalization in Ukraine to be a critical risk factor for colonization of MDR organisms (*7*,*10*). Healthcare practitioners treating citizens of Ukraine need to be cognizant of the increased risk for MDR organism transmission and infection imposed by the conflict in Ukraine and implement appropriate infection control measures to mitigate their spread.

Appendix Additional information for investigation of 6 extensively drug-resistant bacteria in an injured soldier, Ukraine.

## References

[1] Kazmirchuk A, Yarmoliuk Y, Lurin I, Gybalo R, Burianov O, Derkach S, et al. Ukraine’s experience with management of combat casualties using NATO’s four-tier “Changing as Needed” healthcare system. World J Surg. 2022;46:2858–62. 10.1007/s00268-022-06718-3 36070013

[2] Kondratiuk V, Jones BT, Kovalchuk V, Kovalenko I, Ganiuk V, Kondratiuk O, et al. Phenotypic and genotypic characterization of antibiotic resistance in military hospital-associated bacteria from war injuries in the Eastern Ukraine conflict between 2014 and 2020. J Hosp Infect. 2021;112:69–76. 10.1016/j.jhin.2021.03.020 33789157

[3] Higgins PG, Hagen RM, Podbielski A, Frickmann H, Warnke P. Molecular epidemiology of carbapenem-resistant *Acinetobacter baumannii* isolated from war-injured patients from the eastern Ukraine. Antibiotics (Basel). 2020;9:579. 10.3390/antibiotics9090579 32899463 PMC7558915

[4] Wand ME, Bock LJ, Bonney LC, Sutton JM. Mechanisms of increased resistance to chlorhexidine and cross-resistance to colistin following exposure of *Klebsiella pneumoniae* clinical isolates to chlorhexidine. Antimicrob Agents Chemother. 2016;61:e01162–16. 27799211 10.1128/AAC.01162-16 PMC5192135

[5] Simner PJ, Beisken S, Bergman Y, Ante M, Posch AE, Tamma PD. Defining baseline mechanisms of cefiderocol resistance in the Enterobacterales. Microb Drug Resist. 2022;28:161–70. 10.1089/mdr.2021.0095 34619049 PMC8885434

[6] Shaidullina ER, Schwabe M, Rohde T, Shapovalova VV, Dyachkova MS, Matsvay AD, et al. Genomic analysis of the international high-risk clonal lineage *Klebsiella pneumoniae* sequence type 395. Genome Med. 2023;15:9. 10.1186/s13073-023-01159-6 36782220 PMC9926764

[7] Sandfort M, Hans JB, Fischer MA, Reichert F, Cremanns M, Eisfeld J, et al. Increase in NDM-1 and NDM-1/OXA-48-producing *Klebsiella pneumoniae* in Germany associated with the war in Ukraine, 2022. Euro Surveill. 2022;27:2200926. 10.2807/1560-7917.ES.2022.27.50.2200926 36695468 PMC9808319

[8] Giannouli M, Cuccurullo S, Crivaro V, Di Popolo A, Bernardo M, Tomasone F, et al. Molecular epidemiology of multidrug-resistant *Acinetobacter baumannii* in a tertiary care hospital in Naples, Italy, shows the emergence of a novel epidemic clone. J Clin Microbiol. 2010;48:1223–30. 10.1128/JCM.02263-09 20181918 PMC2849555

[9] Del Barrio-Tofiño E, López-Causapé C, Oliver A. Pseudomonas aeruginosa epidemic high-risk clones and their association with horizontally-acquired β-lactamases: 2020 update. Int J Antimicrob Agents. 2020;56:106196. 10.1016/j.ijantimicag.2020.106196 33045347

[10] Zwittink RD, Wielders CC, Notermans DW, Verkaik NJ, Schoffelen AF, Witteveen S, et al.; Dutch CPE and MRSA Surveillance Study Groups. Multidrug-resistant organisms in patients from Ukraine in the Netherlands, March to August 2022. Euro Surveill. 2022;27:2200896. 10.2807/1560-7917.ES.2022.27.50.2200896 36695467 PMC9808315

