# Supplementary material for: Six Extensively Drug-Resistant Bacteria in an Injured Soldier, Ukraine
Source: Emerg Infect Dis. 2023 Aug;29(8):1692–5. doi: 10.3201/eid2908.230567 (PMC10370857; doi:10.3201/eid2908.230567)
Supplement: Appendix — Additional information for investigation of 6 extensively drug-resistant bacteria in an injured soldier, Ukraine. [file 23-0567-Techapp-s1.pdf]

Article DOI: http://doi.org/10.3201/eid2908.230567

EID cannot ensure accessibility for supplementary materials supplied by authors. Readers who have difficulty accessing supplementary content should contact the authors for assistance.

Six Extensively Drug-Resistant Bacteria in an Injured Soldier, Ukraine

Appendix

Appendix Table 1. Antibiotic susceptibility data*

	MIC†				
	**110606**	**110817**	**110818**	**110819**	**110821**
**Antibiotic**	**PSA**	**PSA**	**PSA**	**ACB**	**KPN**
Amikacin	≥64	≥64	32	NA	≥64
Gentamicin	≥16	≥16	8	≥16	≥16
Tobramycin	≥16	≥16	≥16	≥16	≥16
Ampicillin/Sulbactam	NA	NA	NA	≥32	≥32
Cefazolin	≥64	≥64	≥64	≥64	≥64
Cefepime	≥32	≥32	≥32	NA	≥32
Cefotaxime	NA	NA	NA	≥64	≥64
Ceftazidime	≥64	≥64	≥64	≥64	≥64
Ceftazidime/Avibactam	>32	>32	32	32	>32
Ceftolozane/Tazobactam	>8	>8	>8	>8	>8
Imipenem	≥16	≥16	≥16	≥16	≥16
Meropenem	≥16	≥16	≥16	≥16	≥16
Piperacillin/Tazobactam	≥128	≥128	≥128	NA	≥128
Ticarcillin/Clavulanic Acid	≥128	≥128	≥128	NA	NA
Ciprofloxacin	≥4	≥4	≥4	≥4	≥4
Levofloxacin	≥8	≥8	≥8	4	≥8
Tetracycline	NA	NA	NA	2	≥16
Trimethoprim/Sulfameth	NA	NA	NA	≥320	≥320
Colisitin	2	1	1	≤0.25	16
Eravacycline	>8	8	>8	0.25	4
Imipenem	>16	>16	16	>16	>16
Imipenem/Relebactam	>16	>16	2	>16	>16
Meropenem	>8	>8	>8	>8	>8
Meropenem/Vaborbactam	>16	>16	8	>16	>6
Omadacycline	>8	>8	>8	2	>8
Plazomicin	>4	>4	4	>4	>4
Cefiderocol‡	20	21	24	20	8

* PSA, Pseudomonas aeruginosa; ACB, Acinetobacter baumannii, KPN, *Klebsiella pneumoniae*
† Interpretation is based on CLSI (2020) where available. Blue, Resistant; Yellow, Intermediate; Green, susceptible; Orange, Not Interpretable
‡ Performed by Disk diffusion; Results are the Zone of Inhibition expressed in millimeters (mm)

Appendix Table 2. Basic genomic data of Gram-negative strains in this study

MRSN ID	Species	Genome size (Mb)	%G/C	Plasmid Replicons*
110819	*A. baumannii*	3.96	38.9	None
110818	*P. aeruginosa*	6.51	66.0	None
110817	*P. aeruginosa*	6.63	66.1	None
110606	*P. aeruginosa*	6.7	65.9	None
110821	*K. pneumoniae*	5.49	56.7	Col440II, ColRNAI, IncFIB, IncHI1B, IncR

* Plasmid replicons detected using the Plasmid Finder tool (https://bio.tools/PlasmidFinder).
“None” indicates that no replicons present in the database were detected in the strains.
